# RT Slowing to Valid Cues on a Reflexive Attention Task in Children and Young Adults

**DOI:** 10.3389/fpsyg.2018.01324

**Published:** 2018-08-06

**Authors:** Rebecca A. Lundwall, Jason Woodruff, Steven P. Tolboe

**Affiliations:** Psychology Department, Brigham Young University, Provo, UT, United States

**Keywords:** reflexive attention, visuospatial cueing, attentional development, child task characteristics, inhibition of return, visual masking

## Abstract

Peripheral cueing tasks can be used to measure reflexive (automatic) attention. In these tasks, increases in response time or RT (costs) typically follow contralateral (invalid) cues as attention must move from the location of the cue to the target. Reductions in RT (benefits) to a target typically follow ipsilateral (valid) cues because the cue draws attention to where the target will appear. Two exceptions to RT benefits are inhibition of return (IOR) and masking. IOR is the tendency to respond slower to targets that appear in locations attended within the last 200–2000 ms. Masking occurs when the visibility of a target is blocked by another stimulus (e.g., the cue). Herein, we describe two experiments, both using a modified Posner task with “earth rockets” as cues and “alien spaceships” as targets. Cues were equally likely to appear on the left or right side of a display following targets. Participants were instructed to press a left or right key corresponding to a left or right target. In Experiment 1, we obtained data from 203 children (10.58–16.55 years old). We discovered unexpected costs following cues that typically provide RT benefits. In Experiment 2, we explored IOR, masking, and age differences in the occurrence of these costs. We manipulated the cue-target temporal distance (“stimulus onset asynchrony” or SOA) to explore IOR and the cue-target spatial distance to explore masking. We also considered a wider age range. Sixty-three children and 41 young adults participated. Experiment 2 revealed a three-way interaction between SOA, spatial distance, and age. At the shorter SOA (100 ms) and moderate spatial distance, unexpected costs followed valid cues for younger children (7.07–10.15 years old). These costs also occurred in young adults (18.00–23.02 years old) following far distance cues at this SOA. At the longer SOA (200 ms), these costs followed moderate and far cues for younger children and near cues for young adults. Older children (10.31–14.92 years) did not have unexpected costs. We explain the findings in terms of masking, IOR, and possible developmental mechanisms.

## Introduction

Relatively early in the history of modern cognitive psychology, Treisman (1960) described visual attention using a spotlight metaphor. The spotlight metaphor contributed to a theory of selective attention earlier proposed as part of an information processing approach. In his attention model, Broadbent (1958) recognized that we cannot attend to all the information in our environments. Selective attention describes how we “choose” (voluntarily or involuntarily) what we will attend to. The spotlight metaphor suggests that we will tend to pay attention to information that is important for our survival. These selections are important because attention is the foundation of a developmental cascade in which quantitative improvements lead to qualitative shifts in perception, learning, and memory (Fry and Hale, 1996; Rose et al., 2008).

In this paper, we will focus on involuntary selective attention. To distinguish involuntary selective attention from voluntary, we will refer to the former as “reflexive attention,” a term also used by Beane and Marrocco (2004). While involuntary, some aspects of reflexive attention are under voluntary control and interact with voluntary attention (Ristic and Kingstone, 2009). Improved understanding of reflexive attention helps us understand how cognitive processes develop and how the brain works.

To study reflexive attention, Posner (1980) developed the spotlight metaphor into a peripheral cueing task that has been widely used and modified (LaBerge and Brown, 1989; Theeuwes, 1989; Pearson and Lane, 1990; Cheal and Chastain, 2002; Song et al., 2004; Spalek and Hammad, 2004; Thompson et al., 2005; Koenig-Robert and Vanrullen, 2011; Kraft et al., 2011). Posner’s studies of attention also led to a paradigm that included features of reflexive attention such as attention’s ability to engage, disengage, and move locations (Berlucchi, 2006). In the typical Posner reflexive attention task, reduced response times (RTs) are associated with valid (ipsilateral) cues that typically decrease RTs because attention is already at the location where the target appears (Posner and Cohen, 1984; Greenwood et al., 1993). On the other hand, increases in RT typically follow contralateral cues because the spotlight of attention must move from the cue to the target’s location even when there is no eye movement. Attentional movement without eye movement is termed “covert” and is simpler to study than overt attention that includes eye movement (Posner and Cohen, 1984). In Posner’s paradigm, the spotlight metaphor predicts that attended stimuli are processed faster than non-attended stimuli. The fact that it takes longer to respond to a stimulus when attention has been initially engaged farther away from the target than when attention has been engaged near the target (Shulman et al., 1979; Tsal, 1983; Remington and Pierce, 1984) implies that there are attentional costs when attention must move (Mathôt et al., 2010; Kraft et al., 2011).

There are two exceptions that lead to costs when benefits typically appear in Posner’s paradigm. Inhibition of return (IOR) is one exception to the expected RT benefits. IOR refers to the tendency to respond more slowly to targets that appear in recently attended locations within a certain time window (Posner and Cohen, 1984; Maylor et al., 1985; Rafal et al., 1989; Klein, 2000). The time window is usually designated the stimulus onset asynchrony (SOA) and refers to the time from cue onset to target onset. It necessarily includes both cue duration and intertrial interval (gap). The theoretical explanation for slower responding with IOR is typically that IOR prevents perseveration on a single object so that information in other locations can be processed (Posner and Cohen, 1984; Klein, 2000). IOR in adults typically begins about 200–300 ms after cue onset (Posner and Cohen, 1984; Maylor et al., 1985; Rafal et al., 1989; Klein, 2000). After this time window, and lasting until approximately 800–2000 ms, responses tend to be slower. After 800–2000 ms, they are facilitated again (e.g., RTs to targets near where the cue has appeared once again become faster). Clohessy et al. (1991) indicate that IOR develops in infants as perseveration decreases. This supports the idea that IOR is associated with perseveration and may function to reduce it.

Like IOR, masking is another exception to the expectation of RT benefits to valid cues. Breitmeyer and Öğmen (2006) indicates that visual masking occurs whenever the perception of the target is reduced by the presence of another stimulus, referred to as the mask. While there are different types of masking, we will focus on forward masking in which the target follows the cue (the mask) because this is what occurs in our task. This type of masking occurs when two stimuli appear sequentially in essentially the same location and at short cue-target SOAs. In adults, forward visual masking has been described when there is complete or considerable spatial overlap without temporal overlap (Breitmeyer, 1984). Developmental aspects of forward visual masking have not been well explored in children, although auditory masking studies have found that masking decreased as a function of the interstimulus interval (similar to SOA) and age (Soderquist and Schilling, 1992).

Although IOR and masking are attentional patterns that are fairly well understood in adults, IOR is better characterized in infants and adults, and masking is rarely studied in school-aged children. School-aged children might have different IOR and masking parameters because brain maturation is not yet complete. For example, masking may be more powerful or may occur over a larger visual area due to less efficient brain processes (Ross and Ward, 1978; Lawrence et al., 1980; Breitmeyer, 1984; Macchi et al., 2003; Breitmeyer and Ogmen, 2007). While no particular brain regions have been associated with forward masking, primary and secondary visual areas, motion-sensitive regions, and both dorsal and ventral object-sensitive regions have been associated with backward masking (Green et al., 2005). In addition, stronger connectivity from the right intraparietal sulcus to the bilateral fusiform gyrus is associated with a greater effect for attention on the visibility of a target when a mask is employed (Tsubomi et al., 2009).

Like masking, IOR might also be less efficient and occur over a different time course than in adults. IOR has been associated in young adults with the right posterior parietal cortex, right middle occipital cortex, and bilateral superior parietal cortex (Wang et al., 2013). Finally, children’s slower RTs (Sekuler and Mierkiewicz, 1977; Maurer et al., 2005; Brem et al., 2009) might influence the time course of attentional effects.

Some researchers report IOR at different SOAs in children than in adults. For example, MacPherson et al. (2003) found IOR beginning at 570 and 780 ms in younger children (5–10 years) for tasks in which attention is drawn back to central fixation prior to target presentation. They did not find IOR when attention could remain at a cue location prior to target presentation. Likewise, they found IOR beginning at 360 and 780 ms in older children (11–17 years old) for tasks in which attention is drawn back to center prior to target presentation and at 570 ms when attention can remain at a cue location prior to target presentation. However, only a few studies examine how reflexive attention functions in school-age children (Pearson and Lane, 1990; Brodeur and Boden, 2000; Wainwright and Bryson, 2002; Ristic and Enns, 2015; Lundwall and Rasmussen, 2016; Lundwall et al., 2017). In fact, we could find no study (besides our own) in our university library databases (e.g., PsycINFO, Academic Search Premier, MEDLINE) that used typically developing children, a peripheral cueing task, and both valid and invalid cues appearing at chance (50% for two locations). Because this kind of task and the parameters of the possible causes of the costs we found in children are not well established^1^, it seems prudent to explore this gap in the literature.

In the following two experiments, we explore age differences in response to valid cues for which RT benefits are expected. The task included invalid cues, but these are not analyzed because we are focused on a specific question regarding costs following valid cues. First, we describe the unexpected finding in Experiment 1 that children had costs following a valid cue (i.e., it increased their RTs to the subsequent target over a baseline). This is unexpected based on our knowledge of RT benefits in peripheral cueing tasks in adults (Posner, 1980; Greenwood et al., 1993; Mangun and Buck, 1998; Van Damme and Crombez, 2009; Carlisle and Woodman, 2011). In Experiment 2, we explore potential explanations for the findings in Experiment 1 by recruiting a wider age range to examine age-related^2^ differences and by manipulating cue-target spatial distance (near, moderate, and far) and SOA (100 and 200 ms).

In both experiments, we used as baseline a dual-cue condition (two simultaneous cues presented left and right of fixation). Using dual cues as the neutral baseline allows us to retain the influence of a temporal cue (a warning that the target is about to appear) while having a set of cues that do not bias attention to either the right or the left. We acknowledge that some researchers have noted problems with using neutral cues (Jonides and Mack, 1984). For example, the fact that there are two cues on the screen instead of one may change the size of the “spotlight” of attention (Norman, 1968; Posner, 1980) or increase mean luminance compared to a signal cue condition (Gibson, 1996; Pratt et al., 2007; Spehar and Owens, 2012). However, the RT costs and benefits of a dual cue are a useful way to explain attentional processes such as disengaging, moving, and engaging attention, which are key explanations in Posner’s original paradigm (Posner and Cohen, 1984). In addition, many researchers have found useful the calculations of RT costs and benefits as increments and decrements from neutral cues (Akhtar and Enns, 1989; Enns, 1990; Kingstone, 1992; Mcdonald et al., 1999; Klein and Dick, 2002; Wainwright and Bryson, 2002; Li et al., 2003; Mander et al., 2008). In this study, it is especially important to use the unexpected costs following valid cues as the outcome variable because that is the finding from Experiment 1 that we are trying to explain in Experiment 2.

## Experiment 1

We conducted Experiment 1 as part of a longitudinal follow-up study of children whose data were first collected in infancy (Lundwall et al., 2017). The portion reported here examines the direction of effects for the peripheral cueing task. A typical peripheral cueing task (Posner, 1980) includes RT alerting effects, which represent faster RTs (compared to a no-cue condition) that occur due to having a temporal warning that the target is about to appear; RT benefits, which typically represent the faster RTs to valid cues (compared to dual cues) expected from having a temporal and spatial warning that the target is about to appear; and RT costs, which typically represent the slower RTs to invalid cues (compared to dual cues) expected from having a cue contralateral to the location of the subsequent target. Many studies indicate that valid cues are associated with RT benefits and invalid cues are associated with RT costs (Posner and Cohen, 1984; Greenwood et al., 1993) unless the SOA is long, which induces IOR. In Experiment 1, we examined the RT alerting, benefit, and cost scores to determine if they were in the expected direction.

### Materials and Methods

#### Participants

We recruited a general population of children who had participated as infants in a previous study (Dannemiller, 2004). The data for Experiment 1 were collected as part of a follow-up study by researchers at Rice University and the University of Wisconsin–Madison. The data were analyzed at Brigham Young University. The study was approved by Institutional Review Boards at all universities and all work was carried out in accordance with the ethical standards of all universities and with the Declaration of Helsinki, sixth revision. Two hundred and three children participated. All parents gave written informed permission. Children signed assent forms. Two children were excluded for neurological diagnoses, and two children were excluded for uncorrected vision diagnoses. Thus, there were 199 children who completed the peripheral cueing task and whose data were included in statistical analysis. Children were 51% male and ranged in age from 10.58 to 16.55 years (*M* = 12.94 years, *SD* = 1.74).

#### Peripheral Cueing Task and Stimuli

We used a modified Posner reflexive attention task (Posner, 1980) for our peripheral cueing task. We adapted the child task from an adult version that used Xs for cues and small squares for targets (Lundwall et al., 2012). To engage the interest of children while maintaining the ability to measure visual reflexive attention to suddenly appearing stimuli, we designed the task as a game with a backstory and with friendly earth rockets and alien spaceships. These stimuli were designed to be salient and attractive to children. Note that the colors and the proportions of each color in the stimuli were very similar for cues and targets, and the overall size was identical (2.54 cm by 5.08 cm). However, earth rockets (the cues) were oriented vertically and alien spaceships (the targets) were oriented horizontally.

Cues had an inner edge 7.0° from central fixation. Targets had an inner edge 5.7° from fixation. Earth rockets flashed briefly (67 ms). After a brief gap (83 ms), an alien spaceship could appear (93% of trials). The cue duration plus the gap duration yielded an SOA of 150 ms. This SOA is too short to induce eye movements, and attempting to make eye-movements would result in a high error rate.

The cues were equally likely to be valid or invalid indicators of the subsequent target location. Valid cues appeared ipsilateral to where the target would subsequently appear; invalid cues appeared contralateral to where the target would appear. Baseline dual cues involved two simultaneous cues, one on each side of central fixation. In addition, on 7% of trials there were no cues, but targets appeared. We also used catch trials (a cue appeared, but not a target). Because catch trials have no targets, and thus no RTs, they were not used in analyses.

Similar to Kean and Lambert (2003), who used two cue luminances to check for salience effects, we used two cue saturations for the same reason. The use of two different cue saturations^3^ (faded and unfaded, described hereafter as dim and bright, respectively) yielded seven primary measures (i.e., No Cue, Neutral Dim, Neutral Bright, Single Bright Valid, Single Bright Invalid, Single Dim Valid, and Single Dim Invalid). There were 24 trials of each condition intermixed and pseudorandomly presented over the course of the task.

#### Procedure

Participants were tested in a darkened room on a 381 mm × 305 mm Dell monitor with a 60 Hz refresh rate. We maintained viewing distance at 57 cm with a chin rest. We used E-Prime (Psychology Software Tools, Sharpsburg, PA, United States) to present stimuli. During the task, the child would “hit” the alien spaceships by making a left or right key press to the spatially mapped location of the target. The child was instructed to avoid responding to earth-rockets (cues).

Children were told to ignore the cues as much as possible because they did not indicate where the target would appear and would not help their performance. Telling participants to ignore the cues does not bias attention because the cues are, in fact, uninformative (50% of the cues were invalid, although we only analyze responses to valid cues in this paper to seek explanation for the results). If participants attended to the cues, their error rates would be around 50%. Attending to the cues would also have led to more positive benefits rather than to the negative benefits (costs) we describe below.

The participants had 2,000 ms from target onset to respond; however, we only analyzed responses from 200 to 1,000 ms in order to avoid analyzing anticipatory responses or responses after a lapse in attention. When a key press was made, a laser sound played softly (approximately 60 dB), even if the response was incorrect. No sound played if no key press was made. Participants received feedback on each trial as to whether their response had been correct or incorrect. Participants were encouraged to respond as quickly as possible while maintaining high accuracy. Only a participant’s correct responses to valid trials were included in analyses.

As alternate measures of orienting, we have provided the mean of original RTs across trials (not derived from difference scores) for valid and invalid cues and the resulting orienting effect (invalid RT–valid RT). These values cannot be said to control for a participant’s raw RT the way that dual cues do, but they do illustrate the unusual results of Experiment 1 because the orienting effect is usually positive. As seen in **Table 1**, there is no RT advantage for validly cued over invalidly cued trials. Such an advantage, if it had occurred, would be called an “orienting effect” or a “cueing effect” (Fuentes and Campoy, 2008; Marotta et al., 2012). Since it did not occur, we proceed with our analysis using derived (difference) scores.

**Table 1:** RTs (ms) by primary measure.

	Single invalid		Single valid		Orienting effect	
	*Mean*	*SD*	*Mean*	*SD*	*Mean*	*SD*
Bright	390.78	71.14	403.85	71.86	-13.07	52.46
Dim	379.88	75.59	402.44	70.42	-22.57	56.22

#### Analysis

When RTs to valid are subtracted from dual-cues (the baseline condition), positive RT values are expected and indicate faster processing as a “benefit” of the ipsilateral cue. Negative values, on the other hand, are in an unexpected direction and indicate RT slowing compared to the baseline condition. We derived RT difference scores by subtracting between specific pairs of conditions (**Table 2**). This isolates the attentional processes we are interested in because a contralateral cue requires moving attention from the location of the cue to the location of the target. The extra time can be inferred to relate to an extra step in cognitive processing (Donders, 1869/1969). Three of the difference scores are standard in a Posner-type cueing paradigm: RT alerting, benefits, and costs. The use of two different cue saturations yielded six of these three standard, difference scores. For reasons described below, we were only interested in RTs to targets following valid cues between conditions. Other, unrelated results have been previously published (Lundwall et al., 2017). All analyses were performed using SPSS version 24 (IBM Corp.).

**Table 2:** The calculation of difference scores.

Difference score	Primary conditions used in calculation
Alert	No Cue – Dual Cue
Benefit	Dual Cue – Single Valid
Cost	Dual Cue – Single Invalid

*Each difference score had two saturations, bright and dim, yielding a total of six scores*.

A significant difference from zero gives us confidence that the negative direction of the calculated benefit scores were indeed costs and reliable in the sense that they are not likely to have occurred by chance. Based on prior literature (Tassanari et al., 1989; Maruff et al., 1999; Schuller and Rossion, 2004), we expected positive RT benefits. Positive RT benefits indicate that participants usually respond faster to a target that appears after a single, valid cue (Posner, 1980; Jonides and Mack, 1984; Moran et al., 1996; Espeseth et al., 2006).

### Results

#### Descriptives

Before we describe the costs following valid cues, we will provide some basic descriptive statistics. Incorrect trials are excluded from analysis because an incorrect key press would not be a meaningful reflection of RT. However, we examine errors briefly because they can indicate difficulty performing the task. In particular, younger children might make more errors on a task if they found it more difficult than older children or adults. However, analyses of incorrect responses (inaccurate key presses) and RTs out of range (i.e., < 200 ms or >1,000 ms) revealed no significant differences in any type of error either by sex (*P*s > 0.37) or by age (*P*s > 0.19). The difference scores we used in the following analysis were approximately normal (skewness < 1.00) and therefore did not require transformation.

#### Main Analysis: Score Differences

**FIGURE 1:**
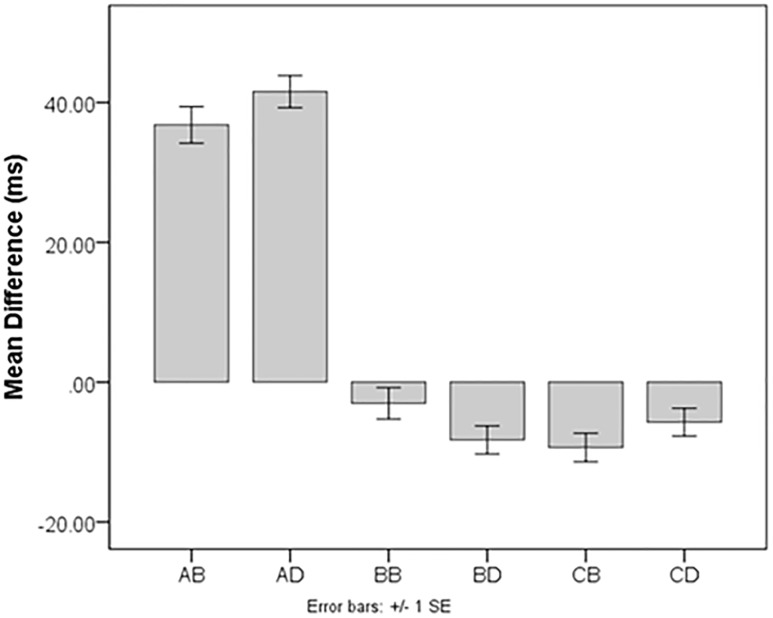
Summary of scores from Experiment 1. Because this study used two saturations (“bright” and “dim”), there are two of each type of score: AB, Alert Bright; AD, Alert Dim; BB, Benefit Bright; BD, Benefit Dim; CB, Cost Bright; CD, Cost Dim. All scores are in the expected directions except for Benefit Bright and Benefit Dim, which were expected to be positive.

Since all we were interested in for this portion of the study was if the scores were in the expected direction (positive or negative), we used a one-sample *t*-test to determine if each score type was significantly different from zero. All difference scores were significantly different from zero: Alert bright *t*(201) = 16.82, *p* < 0.001, *d* = 1.18; alert dim *t*(201) = 20.12, *p* < 0.001, *d* = 1.42; benefit bright *t*(201) = -2.39, *p* = 0.02, *d* = -0.17; benefit dim *t*(201) = -4.67, *p* < 0.001, *d* = -0.33; cost bright *t*(201) = -5.89, *p* < 0.001, *d* = -0.41; and cost dim *t*(201) = -3.08, *p* = 0.01, *d* = -0.22. See **Figure 1** for an illustration. The differences from zero indicate that cues do influence RTs to subsequent targets.

Once the effects of valid and invalid scores were verified, we checked difference scores for the expected direction of effect (i.e., reducing or enhancing RT). RT alerting effects for bright cues (*M* = 36.81, *SD* = 31.10 ms) and dim cues (*M* = 40.35 ms, *SD* = 28.50 ms) were positive, as expected. Mean RT costs were negative, as expected (bright *M* = -10.36, *SD* = 24.99; dim = -5.30, *SD* = 24.47 ms). However, RT benefits were also negative (bright *M* = -4.57, *SD* = 27.13 ms; dim = -7.91, *SD* = 24.18 ms), and this was not expected.

### Summary of Experiment 1

Unlike previous findings with adults (Lundwall et al., 2012), children were slower on average to respond to the target when it was preceded by a valid cue than when it was preceded by simultaneous dual cues that appeared on either side of fixation near the potential target locations (the dual-cues are the baseline condition). RT alerting scores were positive, and costs were negative, both as expected. Costs following valid cues, however, are unexpected because they represent children responding more slowly to a target when it was preceded by a cue that drew their attention to the ipsilateral side.

The SOA for Experiment 1 that produced costs following valid cues in children was 150 ms, which is considered too short to observe IOR for adults (Posner and Cohen, 1984; Maylor et al., 1985; Rafal et al., 1989; Klein, 2000). While classically described IOR seems unlikely to occur at this short SOA, some researchers have argued that IOR does occur at very short SOAs (Danziger and Kingstone, 1999; Dodd and Pratt, 2007). They indicate that facilitation (leading to faster RTs) and inhibition (leading to slower RTs) are two different processes, and their effects can overlap. Brodeur and Boden (2000) argue that most cognitive tasks require both facilitation and inhibition processes that may not be developing at the same rate. Two processes are contrary to the single attentional process that has been proposed by Posner (1980) and Collie et al. (2000). For a single process, which outcome occurs, facilitation or inhibition, depends on time course (SOA). In the two processes perspective, inhibition may begin with the onset of the peripheral cue but is not typically detected at short SOAs due to concurrent facilitation that compensates for early inhibition. This may be especially true for children, whose inhibition tendencies we do not completely understand and whose inhibition might be triggered at different SOAs. Because IOR might be a possible explanation for the costs following valid cues in Experiment 1, in Experiment 2 we tested two SOAs, one shorter and one longer than the SOA used in Experiment 1.

Like IOR, masking is also a possible explanation for the costs following valid cues. In the child task used in Experiment 1, the stimuli appeared in nearly adjacent locations that would not typically be expected to produce masking in adults. It is possible, however, that children experience a “spread of masking” effect. This idea is parallel to spread of inhibition, which has been described by several researchers (Posner and Cohen, 1984; Sereno and Kosslyn, 1991; Kojima and Aiba, 1995; Collie et al., 2000; Spalek and Hammad, 2004; Malinowski et al., 2007). These authors note that inhibition (or increased RT to targets in the same location as previous cues) can spread to several locations and tends to spread over an entire hemifield.

Spreading inhibition tends to occur when the cue stays on display until a key press is made. We could find no studies that address spread of masking in children. However, spread of masking could explain slowed RTs in children when cues and targets are nearer to each other. Cues that are closer to where targets will eventually appear might lead to costs following valid cues if the cues mask the child’s awareness of the subsequent target near that location. In adults, on the other hand, masking would only slow responses when it was substantially overlapping in location with the target. There is little in the literature on the typical development in children of minimal- or non-overlapping forward spatial masking (in which the cue appears before the target), but backward masking studies have suggested age-related changes (Ross and Ward, 1978; Lawrence et al., 1980; Macchi et al., 2003). This suggests that masking might explain the costs following valid cues we found in children in Experiment 1. This hypothesis can be tested in studies using cues (the potential masks) and targets with varying spatial separation. If masking explains the costs following valid cues, we would expect to see more costs following valid cues when the cue is near, fewer when the cue is a moderate distance, and even fewer (or no costs) when the cue is far from where the target subsequently appears.

A third possible explanation for costs following valid cues is that the ability to benefit from a valid cue develops gradually as children increase in their ability to engage attention. If this is true, we might expect some correlation between child age and the benefit scores. However, this is not the case. Child age did not correlate with any peripheral cueing task score except cost following bright, invalid cues (*r* = 0.18, *p* = 0.03). The correlation with cost bright indicates that as children get older, the attentional cost of an invalid cue decreases: they are less distracted by the invalid cue. Age was not correlated (*p* = 0.84) with RT benefits over the age range tested (10.58–16.55 years). Because age is not correlated significantly with RT benefits over the range tested in this study, it is not helpful by itself in explaining why the RT benefits are negative. However, age might participate in interactions with other predictors. For example, inhibition may improve with age such that less irrelevant information is processed (Luna et al., 2004), but this may depend on how close irrelevant information is to relevant information.

We also included sex as a possible explanatory variable because previous literature indicates differences in attention by sex. For example, Continuous Performance Task and Simon Task scores differ by sex (Stoet, 2010; Bush et al., 2015). In addition, Rubia et al. (2010) used an adaptation of the visuo-spatial oddball task in 13- to 38-year-old participants and found differences in brain region activation by sex. The literature also indicates that, by preschool, late-preterm boys experience reduced Differential Ability Scale scores compared to girls (Berry et al., 2013), perhaps suggesting that boys are more vulnerable to a variety of cognitive problems (DiPietro and Voegtline, 2017).

## Experiment 2

We designed Experiment 2 to address possible explanations for the presence of costs following valid cues in Experiment 1. In order to explore all three of the above hypotheses (that IOR, masking, and/or age explain the unexpected costs following valid cues), we designed a study in which task elements varied systematically and compared three age groups of responders. IOR is manipulated by the temporal distance between the cue and the target (Posner and Cohen, 1984). To explore the hypothesis of IOR, we set two SOAs: one longer and one shorter than the SOA used in Experiment 1. Varying SOA allows us to determine if the time course of stimuli presentation is influencing the ability to benefit from a valid cue. To explore the hypothesis of masking, we varied cue distance between the cue and target using near, moderate, and far cues. Varying cue-target spatial distance allows us to determine if masking is influencing the ability to benefit from a cue. By including young adults, we were able to examine age-related differences over the age range of seven to 23 years old.

### Materials and Methods

#### Participants

Children were recruited by placing flyers at child-oriented businesses (such as after-school centers) and by mailing invitations to parents who, according to public birth records, had a child born between 2000 and 2008. Young adults were recruited through the university psychology department’s participant pool. The data for Experiment 2 were collected at Brigham Young University. The study was approved by Brigham Young University Institutional Review Board for Human Subjects, and all work was carried out in accordance with the Declaration of Helsinki, seventh revision. All young adults signed written informed consent. All parents gave written permission, and all children signed written assent. We excluded one participant’s data because he had an uncorrected vision problem and another’s data for having a neurological disorder.

After exclusions, there were 63 children and 41 young adult participants. The young adults ranged in age from 18.00 to 23.02 years (*M* = 19.73, *SD* = 1.46 years), and 46% were male. We divided the children into two age groups: younger and older. There were 32 children in the youngest child age category (44% male) and their ages ranged from age 7.07 to 10.15 years (*M* = 8.62, *SD* = 0.96 years). There were 31 children in the oldest child age category (55% male) and their ages ranged from 10.31 to 14.92 years old (*M* = 12.13, *SD* = 1.48).

#### Peripheral Cueing Task and Stimuli

**FIGURE 2:**
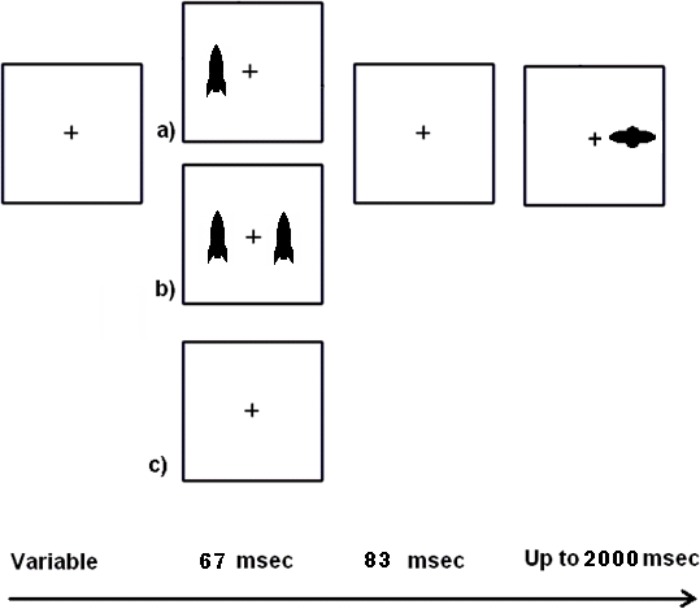
Schematic of the task used in Experiment 1. Following fixation, single, dual, or no cues could appear and were followed by a target on all but catch trials. Single cues could appear on the left or right and were 50% valid for subsequent target location.

The peripheral cueing task used in this study was similar to the one used in Experiment 1 that required child participants to ignore “friendly earth-rockets” and to use a left or right key press to “hit” the “alien spaceships” (**Figure 2**). However, there were two new task manipulations to address the possibilities of IOR and masking. Since Experiment 1 used a 150 ms SOA, Experiment 2 used both a 50 ms shorter SOA (100 ms) and a 50 ms longer SOA (200 ms). IOR has occasionally been found at an SOA of 200 ms (Pratt and Fischer, 2002; Dodd and Pratt, 2007; Zhiguo et al., 2012) or earlier under certain conditions (Danziger and Kingstone, 1999; Dodd and Pratt, 2007). We expected that participants might show more pronounced costs following valid cues at 200 ms if IOR caused the costs following valid cues in Experiment 1. We also address the possibility of masking (Breitmeyer, 1984) by including near, moderate, and far distance cues. We expected that participants would show more costs following valid cues with near than far cues, with moderate distance cues showing a graded effect. This is because the near cues could act as masks that prevent awareness of the target that is presented nearby. This could be especially true for children who may experience spread of masking (i.e., greater spatial extent over which the cue can act as a mask). We included young adult participants to determine whether there are age-related differences in RT benefits from peripheral cueing. For example, costs following valid cues might be most likely in the youngest children, somewhat less likely for older children, and unlikely for young adults. Because IOR and masking are likely to vary across age based on brain development, we expected that there would be interactions with age. For example, if increased RTs to valid trials are observed at 200 ms for both older children and young adults but not for younger children nor at the 100 ms SOA for any participants, then this suggests IOR is an explanation with development as a moderator. A single study in which children and adults experience the same task is necessary to explore these age-related hypotheses.

**FIGURE 3:**
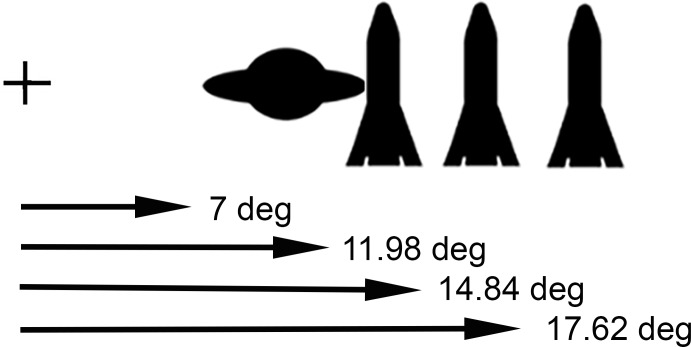
Illustration of the task used in Experiment 2. The target appeared closer to fixation (7° of visual angle) than a preceding cue: near (11.98°), moderate (14.84°), or far (17.62°) distance from fixation.

The experimental conditions were the same as for Experiment 1 except that stimuli were presented with a 75 Hz refresh rate and the cue distances and SOAs were varied as follows. The cues could appear at any of three distances from the fixation cross. “Near” cues had an inner edge 11.98° of visual angle from fixation, “moderate” cues measured 14.84° from fixation, and “far” cues measured 17.62° from fixation. Thus, the targets always appeared closer to fixation than the cues. On near trials, the outside edge of the target overlapped 1° of visual angle with the inside edge of the previously appearing cue (i.e., if they had been on display at the same time). The “moderate” cue had 2° of separation in visual angle from the subsequent target. The “far” cue had 5° of separation from the target (**Figure 3**).

All cues displayed on the screen for 67 ms. After the offset of the cue, there was a variable for either 33 or 133 ms. Following the variable gap, the target appeared with the inner edge 7° left or right from the center fixation cross. Combined with the 67 ms cue presentation, the gap created SOAs of either 100 or 200 ms.

#### Procedure

Unless specified, all experimental procedures were the same as in Experiment 1. However, this experiment was longer to include more trial types. To manage fatigue, we used two identically repeated sessions for a total of 474 trials. There was a mandatory 5-min stretching break halfway through the task. Including paperwork, the total experiment took approximately 45 min. As with Experiment 1, only correct responses between 200 and 1,000 ms after the onset of the target were analyzed.

#### Analysis

We only analyzed trial RT benefits because our primary interest was in determining which of the various manipulations produced costs following valid cues as we observed in Experiment 1. We were also concerned about using highly correlated predictor variables in a regression model (and thus inducing multicollinearity). Previous studies of a similar nature have used factorial ANOVA, but this would not have accounted for the nested nature of the data, in which benefit scores for trial RTs from the same individual are not independent (Hoffman and Rovine, 2007). Multilevel modeling is also especially useful because we are interested in cross-level interactions (Huta, 2014). Multilevel modeling also has other advantages in how missing data are handled, identifying linear and non-linear patterns across time, and handling different numbers of observations per person (Woltman et al., 2012). Some researchers believe that multilevel modeling can help address the speed–accuracy tradeoff that is common in RT tasks (Nieuwenhuizen and Martens, 2016). This is because multilevel analyses model the difference between individuals at one level (how fast they are generally) and RTs on specific trials within that individual (at another level). Multilevel models choose the correct error term when two or more levels are modeled in the same analysis.

To answer our research questions, we cannot use original trial RT because a single trial has no information about whether the participant is faster or slower than their baseline for the cue type. Therefore, we calculated benefits as in Experiment 1. The research question and methods of analysis are as follows: (a) with this task, at what cue distance(s) do we see costs following valid cues? (b) at what SOAs do we see costs following valid cues? and (c) does age interact with cue distance, SOA, or other predictors in the tendency to produce costs following valid cues?

### Results

#### Descriptives

Before we describe our main findings, we will provide some background descriptive statistics. Examining raw RTs, we found the expected age effects (*r* = -0.62, *p* < 0.001), indicating that participants increase in speed (decrease RT) as they age over the range we used. Intercorrelations between raw RTs are shown in Supplementary Table S1. All raw RT values on the peripheral cueing task were correlated (*R*s > 0.84, *P*s < 0.001; see Supplementary Table S1). We expected the correlation of the raw RT scores because they capture similar characteristics such as motor and information processing speed. Intercorrelations between benefit (difference) scores are shown in **Table 3**. Intercorrelations between difference scores are lower, as expected, because an individual’s speed on any valid trial has been subtracted from RTs from the corresponding baseline RTs. Subtracting from a baseline allows each child to become, in a way, their own control. The scores also work well in a regression-based analysis (which we use in the second experiment) since the scores are less correlated and do not induce multicollinearity. Nevertheless, some of the difference scores from the peripheral cueing task are somewhat correlated because they share common features such as cue saturation or validity (e.g., cost bright and benefit bright are more correlated than cost bright and benefit dim) and because they all tap the underlying construct of reflexive attention.

**Table 3:** Intercorrelations of trial benefit scores.

	Near cue benefit at 100 ms	Near cue benefit at 200 ms	Mod cue benefit at 100 ms	Mod cue benefit at 200 ms	Far cue benefit at 100 ms	Far cue benefit at 200 ms
Near cue benefit at 100 ms	1	-0.08	0.42^∗∗^	0.14	-0.10	-0.12
Near cue benefit at 200 ms		1	-0.03	0.22^∗^	-0.07	-0.02
mod cue benefit at 100 ms			1	0.11	-0.18	-0.11
Mod cue benefit at 200 ms				1	-0.16	0.01
Far cue benefit at 100 ms					1	-0.11
Far cue benefit at 200 ms						1

*^∗^Correlation is significant at the 0.05 level (two-tailed). ^∗∗^Correlation is significant at the 0.01 level (two-tailed)*.

As before, we examine errors briefly because they can indicate difficulty performing the task. Error analyses on incorrect responses (inaccurate key presses) and on RTs out of range (i.e., <200 ms or >1,000 ms) revealed no significant differences in any type of error by sex (*P*s > 0.10). However, there were significant differences in error rate by age category for every type of error. Young adults made more “too fast” RT errors [*F*(2, 86) = 9.64, *p* < 0.001]. The youngest child age group had the lowest response side accuracy [*F*(2, 86) = 7.46, *p* = 0.001], the highest “too slow” RT errors [*F*(2, 86) = 26.06, *p* < 0.001], and the highest overall error rate [*F*(2, 86) = 18.31, *p* < 0.001). Another way to look at this is that 97% of the RT errors in children were too slow while 73% of RT errors in young adults were too fast. This could have been because young adults tend to be faster than children, so their RT errors are shifted toward the lower range of their RTs. However, there was no direct correlation between age and error rate (*r* = 0.10, *p* = 0.49). To explore plausible differences, we included error rate as a predictor in the modeling.

#### Main Analysis: Multilevel Modeling

For our main analysis, we tested explanations for the costs following valid cues we found in Experiment 1. We used multilevel modeling in SPSS version 24 (IBM Corp.). Our Level-1 (trial) variables included three cue distances and two SOAs. Level-2 variables included sex and a three-level age category. We used error rate as a level-2 predictor. Trial RT benefits were used as the dependent variable. Note that error rate and trial RT benefits are not negatively correlated as might be expected if there were a speed-accuracy trade off, *r* = 0.33, *p* = 0.02. This is not surprising since RT participants are very unlikely to have time change strategies for valid and dual cues. Also note that there is no significant non-linear relationship between error rate and RT benefits, *F*(1, 617) = 3.45, *p* = 0.06; quadratic is *F*(2, 616) = 2.37, *p* = 0.09; cubic is *F*(3, 615) = 1.85, *p* = 0.14.

We calculated the benefit of each valid trial by subtracting the RT from the mean of the dual-cue, baseline condition for the associated cue-type, SOA, and distance. We used this dependent variable in a multilevel model analysis with all hypothesized predictors (age, error rate, sex, spatial cue distance, and SOA). We iteratively removed the least significant of the most complex interactions (beginning with three-way interactions) one by one and ran each reduced model. This procedure is backward model comparison and is similar to backward ordinary least squares regression. We compared each model to the previous model on Schwarz’s Bayesian information criterion (BIC) and checked for significant improvement using chi-square statistics. Each of the first four models were significantly better than previous model. We stopped creating reduced models when no improvement occurred (Supplementary Table S2).

The final multilevel model includes all five main effect variables, a significant three-way interaction, and all relevant two-way interactions. This model was significantly better than the previous model, χ^2^[2, *N* = 47 625] = 31.52, *p* < 0.001, *f*^2^ = 0.35 (see Supplementary Table S2 for estimates from the multilevel models; see **Table 4** for means and standard errors). We calculated an overall effect size and effect sizes for each predictor according to the suggestions of Selya et al. (2012). As with an ordinary least squares regression, a term that is significant is significant controlling for the other predictors in the model.

**Table 4:** Interaction between SOA, cue distance, and age.

		Benefit (ms)		
Age (years)	Cue distance	SOA (ms)	Mean	*SE*
Younger children	near	100	-4.26	10.08
7.07–10.15		200	8.52	9.84
	mod	100	**-21.74**	9.84
		200	**-27.82**	9.85
	far	100	31.19	9.86
		200	**-13.22**	9.81
Older children	near	100	5.39	9.46
10.31–14.91		200	5.04	9.46
	mod	100	3.12	9.45
		200	-7.01	9.45
	far	100	1.32	9.45
		200	7.69	9.45
Young adults	near	100	-0.54	26.39
18.00–23.02		200	**-27.34**	25.04
	mod	100	-11.59	25.12
		200	23.53	26.15
	far	100	**-28.24**	26.55
		200	38.50	27.55

*All means are adjusted for error rate. Costs following valid cues are bolded*.

**FIGURE 4:**
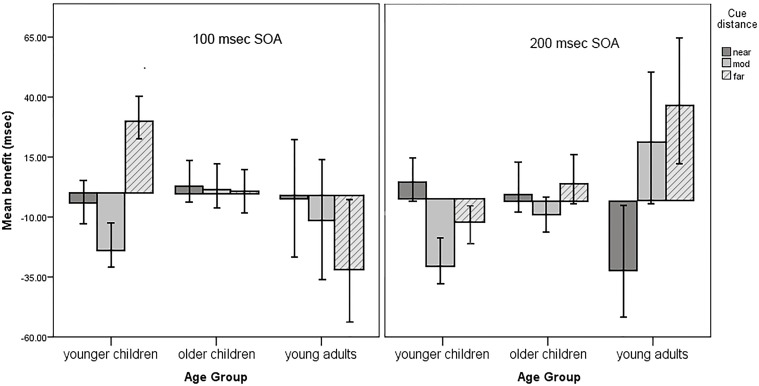
Mean RT benefits, adjusted for error rate. Younger children had significant costs following valid cues at both SOAs for the moderate distance cues. None of the other benefit scores are significantly negative, although younger children approach significant costs following valid cues at the longer SOA for far distance cues. Error bars represent 1 ± the standard error of the mean.

Five terms were significant in the final model. The three-way interaction between age, spatial distance, and SOA was significant, *F*(4, 9072.10) = 3.12, *p* = 0.02, *f*^2^ = 0.002. This is a weak effect size but suggests that younger children had costs following valid cues at moderate cue distances for both SOAs. They also tended to have costs following valid cues at the longer SOA for the far cue distances. Young adults had costs at the far distance for the shorter SOA and at the near distance for the longer SOA. Older children had RT benefits that did not substantially differ from zero at either SOA. See **Figure 4** for an illustration of the three-way interaction.

In addition to the three-way interaction, we found a two-way interaction between sex and cue distance, *F*(2, 9053.87) = 3.16, *p* = 0.04, *f*^2^ = 0.002. In this two-way interaction, there is a linear trend for males having progressively fewer costs following valid cues with increasing cue distance (near *M* = -12.83, *SD* = 188.30 ms; moderate *M* = -7.41, *SD* = 199.04; far *M* = -4.32, *SD* = 195.73 ms). However, females showed positive RT benefits for near (*M* = 13.58, *SD* = 222.10 ms) and far (*M* = 10.82, *SD* = 219.22 ms) cues and costs following moderate cues (*M* = -10.09, *SD* = 233.97 ms).

There was an age by error rate interaction such that the youngest children were most likely to have costs when they also had a high error rate, *F*(2, 9094.11) = 33.91, *p* < 0.001, *f*^2^ = 0.01. Intriguingly, there was also an interaction between error rate and SOA, *F*(1, 9099.21) = 4.41, *p* = 0.04, *f*^2^ = 0.001. While this is a very small effect size, it suggests that costs tend to occur for the longer SOA at higher error rates but not at lower error rates or for the short SOA. The main effect for error rate [*F*(1, 9110.79) = 41.71, *p* < 0.001, *f*^2^ = 0.22] indicates that participants with high error rates were more likely to have benefits following valid cues. No other main effects or interactions were significant. As can be seen in **Figure 4**, there are also linear trends for distance in young adults at both the 100 ms SOA [*F*(2, 2917.41) = 6.40, *p* = 0.002] and the 200 ms SOA [*F*(2, 2911.72) = 9.21, *p* < 0.001]. These effects were tested in follow-up analyses restricting data to young adult participants. The linear trends can be seen occurring in opposite directions across cue distance between the 100 ms SOA and the 200 ms SOA.

### Summary of Experiment 2

Our most basic result is a replication of the lack of benefits to valid cues, as found in Experiment 1. Error rate significantly predicted costs following valid cues such that higher errors were associated with larger costs following valid cues. This occurred despite the fact that we removed trials with errors after calculating each individual’s error rate. The association between error rate and costs cannot be a speed-accuracy trade-off because it involves both higher errors and the failure to benefit from a valid cue. We explore possible explanations in the Section “General Discussion.”

Error rate was the strongest predictor in the model. While the interactions have very small effect sizes, they provide additional insight into how error rate participates with other variables in predicting costs following valid cues. Age-related differences in whether and how much error rate impacts costs seem to imply the development of attentional control mechanisms that can influence even reflexive attention. There were no main effects for SOA, cue-target distance, or age. Since we conducted Experiment 2 to explain costs following valid cues in Experiment 1, we further review the findings of Experiment 2 in the Section “General Discussion.”

## General Discussion

### Overall Summary of Findings

Both Experiments 1 and 2 showed costs following valid cues. This finding is unexpected based on existing literature (Posner, 1980; Greenwood et al., 1993). It is unlikely to be due to chance because similar results were observed in two different samples. The costs following valid cues are interesting on their own because they are not expected from previous research using 50% valid cues. However, few researchers study typically developing children using a peripheral cueing task with both valid and invalid cues appearing at chance. There is no reason to expect that children’s cognitive processes and behavior would match that of adults.

Experiment 2 was a follow-up to explore potential explanations for findings in Experiment 1. In Experiment 2, we found error rate to be the best predictor, and several interactions included error rate in predicting costs following valid cues. Two of these interactions are especially interesting because they involve age-related differences that may relate to developmental processes. We will use our understanding of possible developmental mechanisms to explain our findings in terms of IOR and masking. We also discuss age differences and the influences of error rate.

### Inhibition of Return

Stimulus onset asynchrony between cue and target was not a significant predictor of costs following valid cues. This indicates that IOR alone was not a significant explanatory variable in predicting costs following valid cues. According to the spotlight metaphor (Posner and Cohen, 1984), the dual cue trials in our task should have had slower RTs than the single cue trials (i.e., there should have been positive benefits) for each age group at the 100 ms SOA. However, this was not the case. One possible explanation for the lack of benefits is that both SOAs are too short to reliably induce IOR in children (Brodeur and Enns, 1997; Li et al., 2003; MacPherson et al., 2003). We chose the SOAs we used because they bracketed the SOA that produced costs following valid cues in Experiment 1. Longer SOAs may be useful to explore in future studies with children. The task did seem to be effective in inducing IOR for young adults (**Figure 4**), who had costs following valid cues at the 200 ms SOA. However, this is conjecture as there was no significant age by SOA interaction.

Although there was no age by SOA interaction, there was an age by distance by SOA interaction (with a weak effect size). Those most likely to have costs following valid cues were younger children and young adults. Older children did not demonstrate very much cost following a valid cue. The lack of negative or positive RT benefits in older children combined with the costs following valid cues in younger children and young adults suggests a non-linear developmental process. Several researchers have proposed that there is reorganization of brain structures and networks over the course of development (Gupta et al., 2009; Uhlhaas et al., 2009; Kar et al., 2011). For example, one pattern we see in neuroscience is the tendency to build (or overbuild) capacity and then prune away what is not used. Older childhood might be a transition period between the patterns shown in younger childhood and young adulthood (Liss and Haith, 1970; Lellis et al., 2013).

Imaging studies may offer some insights into developmental processes that might contribute to the lack of benefits in older children. Imaging studies have suggested that attention generally and IOR in particular are handled by the right posterior parietal cortex, including the bilateral superior parietal cortex (Wang et al., 2013). Gray matter volume decreases during adolescence more in the parietal than in other lobes (Mu et al., 2017). The decreases appear to be associated with improved efficiency such as in attentional processes such as IOR (which is an advantage in avoiding perseveration). Given the lack of studies using peripheral cueing tasks with 50% validity in children, studies more fully exploring IOR in children seem to be worth exploring in the future.

### Masking

There was no main effect for cue-target spatial distance (which would have suggested masking). However, there was a sex by distance interaction (with a weak effect size), suggesting that masking might be somewhat more impactful for males than for females. The interaction effect indicates that males were more likely to have costs following near cues, which might mean that they were better at ignoring the cues. Sex differences have been found in other types of attention tasks (Bush et al., 1998; Rubia et al., 2010). One possible sex difference might have to do with video game experience, which is often higher for boys (Marshall et al., 2006; Taverno Ross et al., 2013). This could explain the sex by spatial distance interaction if there is some advantage during video game play to ignore “near” information, such as the heads up displays on video game screens.

Consider the consistent costs following valid cues at a moderate spatial distance across both SOAs for younger children. Recall that masking occurs in adults when two sequential stimuli appear in essentially the same location (MacPherson et al., 2003). Spread of masking might occur with younger children, so costs following valid cues could extend to moderate cues (Breitmeyer, 1984; Sereno and Kosslyn, 1991; Kojima and Aiba, 1995; Malinowski et al., 2007). However, if spread of masking is occurring following moderate distance cues, then we might also expect costs following valid cues following near distance cues. Because that result is not evident here (the benefits are near zero at both SOAs), we suspect that another process is occurring that is overwhelming this effect. One possibility is that facilitation is more dependent on location (Posner and Cohen, 1984; Sereno and Kosslyn, 1991; Kojima and Aiba, 1995; Collie et al., 2000; Spalek and Hammad, 2004; Malinowski et al., 2007) than masking, so masking is occurring at both locations while facilitation occurs more with near than moderate cues (resulting in a near zero benefit). Reduction in the extent of masking might occur with increased age and thus may represent a developmental process.

### Age Differences

There was no main effect for age. However, the three-way interaction including SOA and distance and the two-way interaction with error rate both involved age. Interactions with age imply underlying developmental processes. One possible developmental process that might contribute to age-related differences in attention is increased connectivity (Miller, 1972; Maisto and Baumeister, 1975; Lawrence et al., 1980). Brain connectivity continues to change through young adulthood (Fair et al., 2009; Lopez-Larson et al., 2012). Stronger connectivity from the right intraparietal sulcus to the bilateral fusiform gyrus is associated with a greater improvement in the visibility of a target when a mask is used (Tsubomi et al., 2009). Cortical thinning, white matter volume increases, and increased white matter fiber density (Tamnes et al., 2010) also appear to play a role in improved cognition. Maturation after adolescence involves projection tracts, including prefrontal-striatal connections known to support interhemispheric connectivity. Because brain maturation has been found to unfold in concert with pubertal changes, hormonal influences on white matter development seem likely (Asato et al., 2010). Together, these findings suggest that white matter connectivity may explain some of the age-related differences we found.

### Error Rate

Although we initially considered error rate a covariate, it had the biggest influence on whether participants had costs following valid cues; therefore, we included interactions with error rate. While the main effect for error rate was our largest, interactions are inherently interesting because they suggest what the effect might depend on. For example, the influence of age on costs appears to depend on error rate. Those with higher error rates tended to have more costs following valid cues, and this was especially true in the younger child group. The influence of error rate on costs also appears to depend on SOA, with larger costs with higher error rates at the longer SOA. This suggests more IOR at 200 ms in those with higher error rates. Those with lower error rates might have IOR at longer SOAs that we did not test or simply have less powerful IOR. Another way to look at this is that those with lower error rates were more able to benefit from a valid cue at the 200 ms SOA. Since IOR is controlled by neural efficiency (pruning and increased connectivity) in the parietal lobe, and since neural efficiency develops in adolescence, this implies a developmental influence on who experiences costs following valid cues.

Recall that young adults tended to make more anticipation errors while those in the youngest child age group showed a wider variety of errors and the highest overall error rate. However, there was no direct correlation between age and error rate. Therefore, in explaining costs following valid cues, we cannot simply say that younger children make more errors and more error-prone processing also leads to more costs following valid cues. We also cannot say that the errors were responsible for costs following valid trials. We only analyzed correct trials. Nor can it be visual acuity for far peripheral cues since children benefited from far cues at the shorter SOA. Higher error rate might be related to individual differences in genetic or environmental influences (such as stress) on brain development (Tarullo et al., 2008).

### Overall Trends

There are linear trends apparent at the 200 ms SOA that represent possible developmental patterns. As is shown on the right half of **Figure 4** (200 ms SOA), there is an increasing linear trend for the mean of far cues across our three age groups. This trend indicates that the means are more likely to be negative for younger children and positive for young adults, with older children falling between. A similar pattern existed for moderate cues across age groups: there was negative mean for younger children, a less negative mean for older children, and a positive mean for young adults. Children become increasingly able to process moderate and far cues sufficiently well to benefit from them.

Interestingly, a reverse trend seemed to exist for near cues at this SOA. The mean for young adults was negative, the mean for older children was zero, and for younger children was more positive. This appears to represent the development of masking. This is opposed to the idea of spread of masking and is the first report of the development of masking in the literature. Together, these illustrations of linear trends contribute to the literature on the development of attention because there are no other studies suggesting these trends for the ages we tested using a task like ours.

### Limitations and Future Directions

While we replicated costs following valid cues from Experiment 1 to Experiment 2, further investigation is necessary to fine-tune the developmental explanations behind some of our findings. Our studies indicate that there are developmental processes influencing IOR and likely masking, but it is less clear exactly why these processes occur. We have suggested possible mechanisms through brain development (especially in the parietal lobe) and brain reorganization (including pruning and connectivity) that occurs during puberty. In Experiment 2, an increased sample size might clarify some weak interactions. The sample size for Experiment 1 was 199 participants and for Experiment 2 was 104 participants. The finding of costs following valid cues was replicated, but there are more conditions and interactions in Experiment 2. Multilevel modeling is a powerful tool for detecting main effects, but recent Monte Carlo analyses indicate that cross-level interactions are often underpowered (Mathieu et al., 2012). A larger sample size might confirm our findings and allow exploration of additional explanations that we did not include. For example, it is possible that participants in one age group are employing strategies that participants in another age group do not employ or that participants in one age group have had experiences that have shaped reflexive attention (Ristic and Kingstone, 2009). Future studies should attempt to replicate our findings and consider additional explanations such as these.

## Conclusion

We have reported on a unique developmental study that explores possible reasons for the costs following valid cues that we saw in a sample of children in Experiment 1. Costs following valid cues indicate slower responses to a target (compared to targets following dual cues). We replicated the costs following valid cues in Experiment 2 and demonstrated in a new sample that costs following valid cues for this task are reliable in children and that they vary across age. Younger children are more likely to have costs following valid cues at a moderate distance from the target at both SOAs. There were important linear trends across age at the 200 ms SOA for all cue distances that represent IOR and (possibly) masking. The Posner paradigm with 50% valid peripheral cues and derived RT benefits was helpful in explaining costs following valid cues. Our findings add to the sparse literature on the characteristic responses of children following cues that vary in SOA and spatial distance from a subsequent target. This information increases our basic understanding of the development of IOR and masking. Researchers should consider that both IOR and masking may combine to produce costs following valid cues in their own reflexive attention tasks used with children.

While our findings are primarily experimental and support the view of developmental influences on attention, they may also have implications for the ideal presentation of visual stimuli to children such as in instructional slides or videos. The implications of such findings could include the importance of spatially separated stimuli (so that one stimulus does not impair the processing of another stimulus) and the importance of temporally separated stimuli (to allow sufficient processing of one stimuli before another is presented and interrupts the first). In addition, children may benefit from assistance in removing their attention from an old to a new location. Such modifications might be useful, for example, in children’s educational software. These ideas are suggested not only by our own findings but also by the work of others (Blake and Duffy, 1986; MacPherson et al., 2003).

## Author Contributions

RL designed the studies, collected data, performed statistical analyses, and wrote the paper. JW collected data, assisted in statistical analyses, and helped write the paper. ST modified the task for Experiment 2, assisted in statistical analyses, and helped write the paper.

## Conflict of Interest Statement

The authors declare that the research was conducted in the absence of any commercial or financial relationships that could be construed as a potential conflict of interest.

## Footnotes

1 We refer here to a gap in explanations for unexpected costs in peripheral cueing tasks with 50% valid cues. Many studies use other child tasks (e.g., with central and/or 100% valid cues).

2 Age-related changes can be considered developmental changes in the sense that the passage of time affords opportunities for experience and brain maturation that support maturation of attentional processes (Luna et al., 2004). Nevertheless, we use “age-related” to indicate that we have no developmental mechanism in mind. We use “developmental” to indicate that we have mechanisms in mind.

3 The unfaded stimulus was created first and then the faded stimulus was created by decreasing the former’s saturation in Photoshop. It is not possible to report a single luminance value for colorful stimuli as that value would vary over the image. Taking average luminance does not avoid variability in luminance across a stimulus.
