# Supplementary material for: RT Slowing to Valid Cues on a Reflexive Attention Task in Children and Young Adults
Source: Front Psychol. 2018 Aug 6;9:1324. doi: 10.3389/fpsyg.2018.01324 (PMC6087753; doi:10.3389/fpsyg.2018.01324)
Supplement: Supplementary file 1 [file Table_1.DOCX]

Supplementary Material

RT slowing to valid cues on a reflexive attention task in children and young adults

Rebecca A. Lundwall^*^, Jason Woodruff, Steven P. Tolboe

*** Correspondence:** Corresponding Author: Rebeccca_Lundwall@byu.edu

###### **Supplementary Table 1. Intercorrelations between raw RTs**

|  | NV 100 | NV 200 | NI 100 | NI 200 | MV 100 | MV 200 | MI 100 | MI 200 | FV 100 | FV 200 | FI 100 | FI 200 | DN 100 | DN 200 | DM 100 | DM 200 | DF 100 | DF 200 |
| --- | --- | --- | --- | --- | --- | --- | --- | --- | --- | --- | --- | --- | --- | --- | --- | --- | --- | --- |
| Near Valid (NV) 100 | 1 | .92** | .92** | .95** | .94** | .93** | .94** | .94** | .94** | .94** | .95** | .95** | .90** | .94** | .92** | .94** | .95** | .94** |
| Near Valid (NV) 200 | .92^**^ | 1 | .95^**^ | .93** | .91** | .94** | .88** | .93** | .91** | .92** | .91** | .95** | .93** | .92** | .93** | .92** | .92** | .91** |
| Near Invalid (NI) 100 | .92** | .95** | 1 | .94** | .91** | .92** | .91** | .93** | .93** | .92** | .93** | .94** | .94** | .93** | .95** | .94** | .94** | .92** |
| Near Invalid (NI) 200 | .95** | .93** | .94** | 1 | .93** | .91** | .95** | .94** | .95** | .94** | .94** | .96** | .92** | .96** | .94** | .95** | .96** | .95** |
| Mod Valid (MV) 100 | .94** | .91** | .91** | .93** | 1 | .89** | .89** | .93** | .91** | .90** | .94** | .94** | .87** | .92** | .91** | .93** | .92** | .92** |
| Mod Valid (MV) 200 | .93** | .94** | .92** | .91** | .89** | 1 | .89** | .91** | .89** | .91** | .90** | .92** | .89** | .92** | .89** | .90** | .92** | .90** |
| Mod Invalid (MI) 100 | .94** | .88** | .91** | .95** | .89** | .89** | 1 | .93** | .94** | .93** | .94** | .93** | .89** | .94** | .91** | .92** | .94** | .93** |
| Mod Invalid (MI) 200 | .94** | .93** | .93** | .94** | .93** | .91** | .93** | 1 | .95** | .94** | .94** | .95** | .91** | .94** | .93** | .93** | .93** | .94** |
| Far Valid (FV) 100 | .94** | .91** | .93** | .95** | .91** | .89** | .94** | .95** | 1 | .94** | .95** | .94** | .91** | .96** | .96** | .96** | .94** | .95** |
| Far Valid (FV) 200 | .94** | .92** | .92** | .94** | .90** | .91** | .93** | .94** | .94** | 1 | .93** | .93** | .92** | .95** | .94** | .94** | .94** | .94** |
| Far Invalid (FI) 100 | .95** | .91** | .93** | .94** | .94** | .90** | .94** | .94** | .95** | .93** | 1 | .94** | .90** | .95** | .94** | .96** | .95** | .94** |
| Far Invalid (FI) 200 | .95** | .95** | .94** | .96** | .94** | .92** | .93** | .95** | .94** | .93** | .94** | 1 | .90** | .95** | .93** | .94** | .95** | .93** |
| Dual Near (DN) 100 | .90** | .93** | .94** | .92** | .87** | .89** | .89** | .91** | .91** | .92** | .90** | .90** | 1 | .92** | .94** | .90** | .91** | .89** |
| Dual Near (DN) 200 | .94** | .92** | .93** | .96** | .92** | .92** | .94** | .94** | .96** | .95** | .95** | .95** | .92** | 1 | .94** | .94** | .97** | .94** |
| Dual Mod (DM) 100 | .92** | .93** | .95** | .94** | .91** | .89** | .91** | .93** | .96** | .94** | .94** | .93** | .94** | .94** | 1 | .95** | .94** | .94** |
| Dual Mod (DM) 200 | .94** | .92** | .94** | .95** | .93** | .90** | .92** | .93** | .96** | .94** | .96** | .94** | .90** | .94** | .95** | 1 | .95** | .95** |
| Dual Far (DF) 100 | .95** | .92** | .94** | .96** | .92** | .92** | .94** | .93** | .94** | .94** | .95** | .95** | .91** | .97** | .94** | .95** | 1 | .92** |
| Dual Far (DF) 200 | .94** | .91** | .92** | .95** | .92** | .90** | .93** | .94** | .95** | .94** | .94** | .93** | .89** | .94** | .94** | .95** | .92** | 1 |
