# Supplementary material for: RT Slowing to Valid Cues on a Reflexive Attention Task in Children and Young Adults
Source: Front Psychol. 2018 Aug 6;9:1324. doi: 10.3389/fpsyg.2018.01324 (PMC6087753; doi:10.3389/fpsyg.2018.01324)
Supplement: Supplementary file 2 [file Table_2.DOCX]

Supplementary Material

RT slowing to valid cues on a reflexive attention task in children and young adults

Rebecca A. Lundwall^*^, Jason Woodruff, Steven P. Tolboe

*** Correspondence:** Corresponding Author: Rebeccca_Lundwall@byu.edu

###### **Supplementary Table 2. Estimates from Models of the Predictors of Unexpected Costs**

|  |  | Empty | | Full | | | Remove worst 3-way | | | | | Remove next 3-way | | | | Remove last NS 3-way | | | |
| --- | --- | --- | --- | --- | --- | --- | --- | --- | --- | --- | --- | --- | --- | --- | --- | --- | --- | --- | --- |
|  | Parameter | Mean | SE | Mean | | SE | Mean | | | SE | | Mean | SE | | | Mean | | SE | |
|  |  | Fixed | | | | | | | | | | |  |  |  |  |  |  |  |
| Intercept |  | -31 | 4.07 | 29.07 | 31.29 | | | 34.57 | 30.44 | | 33.39 | | | 30.35 | 28.58 | | 28.67 | | |
| Level 1 (trial-specific) | | |  |  |  | | |  |  | |  | | |  |  | |  | | |
|  | Distance | (near) |  | -60.80 | 40.25 | | | -61.83 | 40.23 | | -62.35 | | | 40.05 | -54.87 | | 36.27 | | |
|  |  | (mod) |  | -25.81 | 40.60 | | | -26.06 | 40.60 | | -21.75 | | | 40.39 | -15.14 | | 37.00 | | |
|  |  | (far) |  | 0^a^ | 0.00 | | | 0^a^ | 0.00 | | 0^a^ | | | 0.00 | 0^a^ | | 0.00 | | |
|  | SOA | (100 msec) |  | -45.75 | 40.20 | | | -56.54 | 37.60 | | -65.35 | | | 37.72 | -54.66 | | 37.28 | | |
|  |  | (200 msec) |  | 0^a^ | 0.00 | | | 0^a^ | 0.00 | | 0^a^ | | | 0.00 | 0^a^ | | 0.00 | | |
| Level 2 (participant- and cross-level) | | | |  |  | | |  |  | |  | | |  |  | |  | | |
|  | Error rate | | | -65.17*** | 41.29 | | | -64.83 | 41.28 | | -64.98 | | | 41.27 | -66.54 | | 41.16 | | |
|  | Sex (male) | | | 13.33 | 48.68 | | | -4.47 | 42.67 | | -0.67 | | | 41.95 | 14.62 | | 27.40 | | |
|  | Age (youngest) | | | -26.61 | 33.33 | | | -33.71 | 32.35 | | -33.92 | | | 32.35 | -29.90 | | 30.39 | | |
|  | (middle) | | | -15.22 | 33.73 | | | -19.72 | 32.67 | | -20.47 | | | 32.64 | -13.75 | | 30.52 | | |
|  | (oldest) | | | 0^a^ | 0.00 | | | 0^a^ | 0.00 | | 0^a^ | | | 0.00 | 0^a^ | | 0.00 | | |
|  | Age * Error rate (youngest) | | | 268.60 | 42.12 | | | 267.85 | 42.12 | | 268.19 | | | 42.11 | 269.50 | | 42.00 | | |
|  | (middle) | | | 163.10 | 43.00 | | | 162.46 | 42.99 | | 162.88 | | | 42.99 | 164.43 | | 42.88 | | |
|  | (oldest) | | | 0^a^ | 0.00 | | | 0^a^ | 0.00 | | 0^a^ | | | 0.00 | 0^a^ | | 0.00 | | |
|  | Error rate * SOA (100 msec) | | | 33.92 | 16.42 | | | 34.08 | 16.42 | | 34.09 | | | 16.42 | 34.49 | | 16.43 | | |
|  | (200 msec) | | | 0^a^ | 0.00 | | | 0^a^ | 0.00 | | 0^a^ | | | 0.00 | 0^a^ | | 0.00 | | |
|  | Distance * SOA (near, 100 msec) | | | 90.57 | 51.80 | | | 92.33 | 51.75 | | 93.49 | | | 51.19 | 93.34 | | 51.16 | | |
|  | (near, 200 msec) | | | 0^a^ | 0.00 | | | 0^a^ | 0.00 | | 0^a^ | | | 0.00 | 0^a^ | | 0.00 | | |
|  | (mod, 100 msec) | | | 36.12 | 51.68 | | | 36.64 | 51.68 | | 28.22 | | | 51.05 | 28.85 | | 51.04 | | |
|  | (mod, 200 msec) | | | 0^a^ | 0.00 | | | 0^a^ | 0.00 | | 0^a^ | | | 0.00 | 0^a^ | | 0.00 | | |
|  | (far, both SOAs) | | | 0^a^ | 0.00 | | | 0^a^ | 0.00 | | 0^a^ | | | 0.00 | 0^a^ | | 0.00 | | |
|  | Sex * distance (male, near) | | | 3.77 | 57.49 | | | 7.23 | 57.32 | | 8.94 | | | 55.95 | -15.02 | | 12.84 | | |
|  | (male, mod) | | | 52.85 | 56.39 | | | 52.96 | 56.40 | | 38.70 | | | 57.74 | 17.19 | | 12.76 | | |
|  | (male, far) | | | 0^a^ | 0.00 | | | 0^a^ | 0.00 | | 0^a^ | | | 0.00 | 0^a^ | | 0.00 | | |
|  | Age * distance (youngest, near) | | | 94.81 | 42.88 | | | 95.87 | 42.86 | | 95.35 | | | 42.85 | 82.99 | | 38.40 | | |
|  | (youngest, mod) | | | -8.53 | 43.16 | | | -8.33 | 43.17 | | -7.33 | | | 43.16 | -6.80 | | 39.11 | | |
|  | (youngest, far) | | | 0^a^ | 0.00 | | | 0^a^ | 0.00 | | 0^a^ | | | 0.00 | 0^a^ | | 0.00 | | |
|  | (middle, near) | | | 61.58 | 43.30 | | | 62.56 | 43.28 | | 61.51 | | | 43.17 | 58.43 | | 38.34 | | |
|  | (middle, mod) | | | 2.96 | 43.57 | | | 3.18 | 43.57 | | 6.20 | | | 43.48 | -10.44 | | 39.05 | | |
|  | (middle, far) | | | 0^a^ | 0.00 | | | 0^a^ | 0.00 | | 0^a^ | | | 0.00 | 0^a^ | | 0.00 | | |
|  | (oldest, all distances) | | | 0^a^ | 0.00 | | | 0^a^ | 0.00 | | 0^a^ | | | 0.00 | 0^a^ | | 0.00 | | |
|  | Sex * SOA (male, 100 msec) | | | -42.66 | 47.62 | | | -9.22 | 18.07 | | -16.34 | | | 10.46 | -16.23 | | 10.46 | | |
|  | (male, 200 msec) | | | 0^a^ | 0.00 | | | 0^a^ | 0.00 | | 0^a^ | | | 0.00 | 0^a^ | | 0.00 | | |
|  | Age * SOA (youngest, 100 msec) | | | 89.92 | 42.49 | | | 103.88 | 39.44 | | 104.35 | | | 39.43 | 104.78 | | 39.42 | | |
|  | (youngest, 200 msec) | | | 0^a^ | 0.00 | | | 0^a^ | 0.00 | | 0^a^ | | | 0.00 | 0^a^ | | 0.00 | | |
|  | (middle, 100 msec | | | 47.53 | 42.77 | | | 56.27 | 39.46 | | 57.84 | | | 39.33 | 58.25 | | 39.33 | | |
|  | (middle, 200 msec) | | | 0^a^ | 0.00 | | | 0^a^ | 0.00 | | 0^a^ | | | 0.00 | 0^a^ | | 0.00 | | |
|  | (oldest, both SOAs) | | | 0^a^ | 0.00 | | | 0^a^ | 0.00 | | 0^a^ | | | 0.00 | 0^a^ | | 0.00 | | |
|  | Age * sex (youngest, male) | | | -42.79 | 50.80 | | | -21.00 | 44.08 | | -21.27 | | | 44.09 | -34.56 | | 27.59 | | |
|  | (middle, male) | | | -24.89 | 50.52 | | | -8.95 | 43.83 | | -9.19 | | | 43.84 | -27.96 | | 27.41 | | |
|  | (oldest, male) | | | 0^a^ | 0.00 | | | 0^a^ | 0.00 | | 0^a^ | | | 0.00 | 0^a^ | | 0.00 | | |
|  | Sex * distance * SOA (male, near, 100 msec) | | | 5.91 | 25.67 | | | 5.74 | 25.67 | | [removed] | | |  |  | |  | | |
|  | (male, mod, 100 msec) | | | -26.58 | 25.52 | | | -26.76 | 25.52 | |  | | |  |  | |  | | |
|  | (male, far, 100 msec) | | | 0^a^ | 0.00 | | | 0^a^ | 0.00 | |  | | |  |  | |  | | |
|  | Age*distance*SOA (youngest, near, 100 msec) | | | -150.23** | 54.63 | | | -152.04** | 54.58 | | -151.12** | | | 54.54 | -150.56* | | 54.51 | | |
|  | (youngest, near, 200 msec) | | | 0^a^ | 0.00 | | | 0^a^ | 0.00 | | 0^a^ | | | 0.00 | 0^a^ | | 0.00 | | |
|  | (youngest, mod, 100 msec) | | | -62.08 | 54.40 | | | -62.50 | 54.39 | | -64.72 | | | 54.36 | -65.34 | | 54.36 | | |
|  | (youngest, mod, 200 msec) | | | 0^a^ | 0.00 | | | 0^a^ | 0.00 | | 0^a^ | | | 0.00 | 0^a^ | | 0.00 | | |
|  | (youngest, far, 100 msec) | | | 0^a^ | 0.00 | | | 0^a^ | 0.00 | | 0^a^ | | | 0.00 | 0^a^ | | 0.00 | | |
|  | (youngest, far, 200 msec) | | | 0^a^ | 0.00 | | | 0^a^ | 0.00 | | 0^a^ | | | 0.00 | 0^a^ | | 0.00 | | |
|  | (middle, near, 100 msec) | | | -84.92 | 54.68 | | | -86.58 | 54.63 | | -84.60 | | | 54.25 | -65.34 | | 54.36 | | |
|  | (middle, near, 200 msec) | | | 0^a^ | 0.00 | | | 0^a^ | 0.00 | | 0^a^ | | | 0.00 | 0^a^ | | 0.00 | | |
|  | (middle, mod, 100 msec) | | | -3.92 | 54.43 | | | -4.33 | 54.43 | | -10.58 | | | 54.12 | -11.21 | | 54.11 | | |
|  | (middle, mod, 200 msec) | | | 0^a^ | 0.00 | | | 0^a^ | 0.00 | | 0^a^ | | | 0.00 | 0^a^ | | 0.00 | | |
|  | (middle, far, 100 msec) | | | 0^a^ | 0.00 | | | 0^a^ | 0.00 | | 0^a^ | | | 0.00 | 0^a^ | | 0.00 | | |
|  | (middle, far, 200 msec) | | | 0^a^ | 0.00 | | | 0^a^ | 0.00 | | 0^a^ | | | 0.00 | 0^a^ | | 0.00 | | |
|  | (oldest, all distances, both SOAs) | | | 0^a^ | 0.00 | | | 0^a^ | 0.00 | | 0^a^ | | | 0.00 | 0^a^ | | 0.00 | | |
|  | Age * sex * distance (youngest, male, near) | | | -33.74 | 59.35 | | | -37.25 | 59.19 | | -36.32 | | | 59.19 | [removed] | |  | | |
|  | (youngest, male, mod) | | | -4.48 | 57.97 | | | -4.49 | 57.97 | | -3.57 | | | 57.97 |  | |  | | |
|  | (youngest. male, far) | | | 0^a^ | 0.00 | | | 0^a^ | 0.00 | | 0^a^ | | | 0.00 |  | |  | | |
|  | (middle, male, near) | | | -13.72 | 58.96 | | | -17.10 | 58.80 | | -15.94 | | | 58.79 |  | |  | | |
|  | (middle, male, mod) | | | -40.66 | 57.63 | | | -40.69 | 57.63 | | -39.80 | | | 57.63 |  | |  | | |
|  | (middle, male, far) | | | 0^a^ | 0.00 | | | 0^a^ | 0.00 | | 0^a^ | | | 0.00 |  | |  | | |
|  | (oldest, male, all distances) | | | 0^a^ | 0.00 | | | 0^a^ | 0.00 | | 0^a^ | | | 0.00 |  | |  | | |
|  | Age * sex * SOA (youngest, male, 100 msec) | | | 41.46 | 47.84 | | | [removed] |  | |  | | |  |  | |  | | |
|  | (youngest, male, 200 msec) | | | 0^a^ | 0.00 | | |  |  | |  | | |  |  | |  | | |
|  | (middle, male, 100 msec) | | | 29.72 | 47.53 | | |  |  | |  | | |  |  | |  | | |
|  | (middle, male, 200 msec) | | | 0^a^ | 0.00 | | |  |  | |  | | |  |  | |  | | |
|  | (oldest, male, both SOAs) | | |  |  | | |  |  | |  | | |  |  | |  | | |
|  |  | Covariance | | | | | | | | | | |  | | | | | |  |
| Residual | | 44520.36 | 525.63 | 59932.34 | | 891.29 | 59936.90 | | | 891.36 | | 59948.54 | 891.53 | | | 59982.63 | | 892.04 | |
| Number of parameters | | 3 |  | 38 | |  | 36 | | |  | | 34 |  | | | 30 | |  | |
| Swartz’s BIC | | 195819.01 |  | 126460.20 | |  | 126442.83 | | |  | | 126426.44 |  | | | 126394.92 | |  | |

***Significant at *p* < .001. ** Significant at *p* < 0.01. * Significant at *p* < 0.05. Significant values indicate that the estimate is different from zero.

The Table represents backwards modeling, similar to backwards regression. Models were run until all parameters were either significant or part of a higher-level interaction. According to Swartz’s Bayesian Information Criteria (BIC), the last model represented is the best of the models tested. This difference is significant (*p* < .001).

^a^ This parameter has been set to zero.

NS = the parameter was included but not significant. The breakdown of parameters is included if they contributed to a higher-level interaction, even if they were not significant.

[removed] = the parameter was not included in the particular model described.
